# Skin Conductance as a Viable Alternative for Closing the Deep Brain Stimulation Loop in Neuropsychiatric Disorders

**DOI:** 10.3389/fnins.2019.00780

**Published:** 2019-08-07

**Authors:** Dilranjan S. Wickramasuriya, Md. Rafiul Amin, Rose T. Faghih

**Affiliations:** Computational Medicine Laboratory, Department of Electrical and Computer Engineering, University of Houston, Houston, TX, United States

**Keywords:** skin conductance (SC), deep brain stimulation (DBS), deconvolution analysis, arousal, state-space (SS) representation

## Abstract

Markers from local field potentials, neurochemicals, skin conductance, and hormone concentrations have been proposed as a means of closing the loop in Deep Brain Stimulation (DBS) therapy for treating neuropsychiatric and movement disorders. Developing a closed-loop DBS controller based on peripheral signals would require: (i) the recovery of a biomarker from the source neural stimuli underlying the peripheral signal variations; (ii) the estimation of an unobserved brain or central nervous system related state variable from the biomarker. The state variable is application-specific. It is emotion-related in the case of depression or post-traumatic stress disorder, and movement-related for Parkinson's or essential tremor. We present a method for closing the DBS loop in neuropsychiatric disorders based on the estimation of sympathetic arousal from skin conductance measurements. We deconvolve skin conductance via an optimization formulation utilizing sparse recovery and obtain neural impulses from sympathetic nerve fibers stimulating the sweat glands. We perform this deconvolution via a two-step coordinate descent procedure that recovers the sparse neural stimuli and estimates physiological system parameters simultaneously. We next relate an unobserved sympathetic arousal state to the probability that these neural impulses occur and use Bayesian filtering within an Expectation-Maximization framework for estimation. We evaluate our method on a publicly available data-set examining the effect of different types of stress on peripheral signal changes including body temperature, skin conductance and heart rate. A high degree of arousal is estimated during cognitive tasks, as are much lower levels during relaxation. The results demonstrate the ability to decode psychological arousal from neural activity underlying skin conductance signal variations. The complete pipeline from recovering neural stimuli to decoding an emotion-related brain state using skin conductance presents a promising methodology for the ultimate realization of a closed-loop DBS controller. Closed-loop DBS treatment would additionally help reduce unnecessary power consumption and improve therapeutic gains.

## Introduction

Deep Brain Stimulation (DBS) is a type of therapy involving the application of high frequency electrical stimulation, usually at ~130 Hz, to specific anatomical structures deep within the brain (Oluigbo et al., 2012; Carron et al., 2013). While the precise mechanics of the therapy are yet to be fully understood, it has been hypothesized that DBS mimics the effect of ablative lesions without causing any tissue damage (Dostrovsky and Lozano, 2002). A second hypothesis suggests that stimulation from the implanted electrodes modulates electrical circuit activity within dysfunctional brain regions (Oluigbo et al., 2012; Cleary et al., 2015). DBS has been approved by the Food and Drug Administration (FDA) for the treatment of Parkinson's disease and essential tremor in the United States. Humanitarian device exemptions have also been granted by the FDA for the use of DBS in the treatment of severe obsessive compulsive disorder and dystonia (Grahn et al., 2014). Meanwhile, the therapy has also been investigated as a treatment option for a host of other medical conditions including major depression (Puigdemont et al., 2012; Merkl et al., 2013), chronic pain (Boccard et al., 2015; Lempka et al., 2017), drug-resistant epilepsy (Vesper et al., 2007; Fisher et al., 2010), anorexia nervosa (Lipsman et al., 2013; Wu et al., 2013), and substance abuse (Zhou et al., 2011; Müller et al., 2013).

Commercially-available DBS systems currently function in an open loop manner. In open-loop DBS, stimulation is delivered continuously until manually re-adjusted. In contrast, a closed-loop DBS (CLDBS) system automatically adjusts stimulation parameters based on sensor feedback recorded from the patient (Herron et al., 2017). The feedback signal is usually based on a symptom-related biomarker (Bouthour et al., 2019). Open-loop systems can require multiple post-operative visits in the months following surgery (Grahn et al., 2014). During visits, different parameters of the electrical stimulation including frequency, amplitude, and pulse width are adjusted for improving therapeutic benefit (Bronstein et al., 2011). Manual adjustment of the parameters in a trial-and-error fashion is time consuming. It is also challenging to explore the complete stimulation parameter space during brief patient visits. Moreover, open-loop DBS systems apply stimulation even if not strictly required. Consider, for instance, two common movement disorders—essential tremor and Parkinson's disease. Motor symptoms for both disorders include rhythmic involuntary movements (tremors). In essential tremor, the tremors occur during volitional movement (Plumb and Bain, 2007) and stimulation may be unnecessary when a patient is not using an affected limb (Herron et al., 2017). The tremors occur at rest in Parkinson's (Chou et al., 2011). However, motor symptoms can fluctuate continually (Rosin et al., 2011; Little et al., 2013). Evidence suggests that local field potential (LFP) β-band oscillations in the subthalamic nucleus correlate with motor impairment in Parkinson's (Little and Brown, 2012). CLDBS systems switching on control based on LFP threshold crossings were shown to have superior performance in treating Parkinson's patients and had substantial gains in reducing stimulation time (Little et al., 2013, 2016). The effectiveness of CLDBS over an open-loop stimulation in Parskinson's was also shown in non-human primates (Rosin et al., 2011). CLDBS systems have thus arisen gradually to eliminate part of the inefficiencies of their open-loop predecessors.

In a recent work describing a theoretical framework for the design of a CLDBS system for treating chronic pain, Shirvalkar et al. (2018) point out two important elements of closing the loop: (i) the extraction of an accurate, relevant, and timely biomarker of the underlying state variable of interest; (ii) a control-theoretic (e.g., state-space) representation of the system relating the biomarker to the unobserved state variable. In their specific application, they suggest using LFPs from the somatosensory cortex, the dorsal anterior cingulate cortex and the orbitofrontal cortex for tracking a multidimensional pain state. Others have similarly suggested neurochemical biomarkers, skin conductance features, and hormone concentrations as a means of feedback for treating a broad range of neuropsychiatric disorders (Grahn et al., 2014; Bina and Langevin, 2018). Following the suggestion of Shirvalkar et al. (2018), we present a proof-of-principle state-space framework that can be used for CLDBS therapy.

DBS has recently emerged as a potentially successful treatment option for patients diagnosed with post-traumatic stress disorder (PTSD) (Koek et al., 2014; Langevin et al., 2016). PTSD is a type of psychiatric disorder that can occur in patients who have experienced traumatic or stressful events in the past. Distressing memories or dreams often persist long after the event (Jetly et al., 2015). Symptoms of PTSD include changes in psychological arousal, reactivity and mood, and are evidenced by factors such as hypervigilance and exaggerated startle responses (American Psychiatric Association, 2013). This state of hyperarousal or hypervigilance in PTSD has been noted in multiple studies (Woodward et al., 2000; Risser et al., 2006; Hellmuth et al., 2012). While the method we present here could find broader applicability to a range of neuropsychiatric disorders, it seems particularly suited to address PTSD with its hyperarousal symptoms.

Bina and Langevin (2018) suggest the possibility of monitoring skin conductance changes as a potential biomarker in a CLDBS system for treating PTSD. Sympathetic nerve fibers innervate the sweat glands (Low, 2012). Consequently, changes in the conductivity of the skin owing to perspiration provide a measure of sympathetic drive or arousal (Critchley et al., 2002). Heightened responsivity in terms of skin conductance has been noted in PTSD patients compared to controls (Orr and Roth, 2000). In a three-group study of Vietnam combat veterans with PTSD, psychiatric Vietnam combat veterans, and psychiatric non-combat Vietnam-era veterans, McNally et al. (1987) reported that PTSD subjects had the largest skin conductance responses (SCRs) in response to combat-related words. In a similar study comprising of Vietnam combat veterans with PTSD, Vietnam combat veterans, and non-combat controls, Goldfinger et al. (1998) reported that PTSD veterans had the highest baseline skin conductance levels. In an affect-toned Rorschach test conducted in the same study, arousal, as measured by skin conductance, was highest in the PTSD group as well. Pole (2007) also noted higher skin conductance baselines, larger SCRs and slower skin conductance habituation in startle and trauma-cue studies in PTSD patients in a meta-analysis study of adults with and without PTSD. Skin conductance changes also occur in depression. Ward and Doerr (1986) measured skin conductance in patients with depression, parents of firstborn one- to three-month old infants and control subjects, and found that depressed patients had significantly lower skin conductance levels than the other groups. Lin et al. (2011) examined the effects of stress and depression using a series of physiological measures. Participants were first categorized into the normal, low-risk and high-risk depression groups and were assigned to one of two stress treatments. Percentage change in skin conductance between baseline and during the stress treatment periods were significantly dependent on and correlated positively with depression. Both PTSD and depression are potential candidates for DBS therapy when other treatment options have been exhausted. Skin conductance additionally has the advantage of being easily measured with wearable devices such as the Empatica E4 (Koskimäki et al., 2017). Wearable devices afford convenience, seamless integration into clothing and do not involve the risks of surgically implanted sensors.

**Figure 1:**
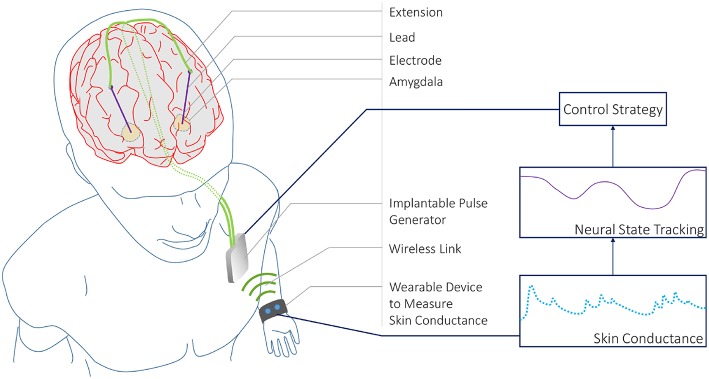
A CLDBS System based on Skin Conductance Measurements. A conceptual CLDBS architecture for treating neuropsychiatric disorders based on tracking a neural state from peripheral skin conductance measurements. Koek et al. (2014) and Langevin et al. (2016) applied stimulation to the amygdala in treatment-refractory PTSD patients. The amygdala plays an important role in emotion processing. In particular, the basolateral nucleus of the amygdala contains cells that are responsive to both fear acquisition and fear extinction (Lüthi and Lüscher, 2014). Koek et al. (2014) and Langevin et al. (2016) targeted this area owing to the partially dysfunctional fear extinction mechanism for trauma-related cues in PTSD patients.

We develop a state-space model to track an unobserved sympathetic arousal state from skin conductance measurements. The relationship between arousal and skin conductance has been attested to in multiple studies (Boucsein, 2012). Individual SCRs are a notable feature in a skin conductance signal. SCRs accompany psychologically arousing stimuli as the skin's conductivity increases momentarily. We relate arousal to the rate at which SCRs occur. Current methods for detecting SCRs in a skin conductance signal rely on detecting peaks above a threshold set between 0.01 and 0.05 μS (Benedek and Kaernbach, 2010). Inter-subject variability in skin conductance signals is a known phenomena (Dawson et al., 2007). We therefore use a deconvolution strategy for extracting the physiological parameters related to sweat secretion for each individual and detect neural impulses to the eccrine sweat glands that generate the SCRs rather than relying on heuristic peak detection. The following section describes our two-part methodology. We first describe the deconvolution approach that utilizes its own state-space formulation for detecting neural impulses based on sweat diffusion and evaporation dynamics. We next describe the state-space formulation for the CLDBS system that relates the probability of neural impulses to a latent sympathetic arousal state. We present our results thereafter and finally conclude with a discussion of our results, and how our methodology could be used in an experimental CLDBS prototype (e.g., such as in the conceptual architecture depicted in Figure 1).

## 1. Materials and Methods

### 1.1. Data

We use the Non-EEG Dataset for Assessment of Neurological Status (Birjandtalab et al., 2016). The data is publicly available through the PhysioNet database (Goldberger et al., 2000). The data-set contains skin conductance recordings from 20 healthy college students who were exposed to physical, emotional, and cognitive stress during three different time periods. Skin conductance was recorded using the wrist-worn Affectiva Q Curve device. Skin conductance can be contaminated by noise sources such as motion artifacts, range saturation and amplification factor changes (Boucsein, 2012). Many of the signals had to be discarded owing to motion artifact contamination and noise due to bad skin contact. Hence, we only used the data from six subjects. We re-labeled the original subject numbers with corresponding participant numbers (Table 1). The physical, cognitive, and emotional stress periods each lasted 5 min and were interspersed by 5 min intervals of relaxation. Subjects were made to stand, walk and jog during the physical stress part of the experiment. We excluded data from this portion of the experiment and focus only on the psychological aspects. The cognitive stress portion consisted of two separate tasks. In the first task, subjects had to count backwards in 7's beginning at 2,485 for 3 min and then perform the Stroop test for a further 2 min. In a Stroop test, a subject is shown a word denoting a color and is asked to read it out. However, the color in which the text is written may not necessarily correspond to what it means. A buzzer notified subjects of any errors they made. Emotional stress was induced by means of a horror movie clip. The authors of the data-set noted that many of the volunteers participating in the experiment showed a stress response that was visible to the experiment administrator while they were just being given instructions regarding the cognitive tasks. Hence, they categorized the 40 s interval just prior to the counting task and the Stroop test as a stress period.

**Table 1:** Subject information for selected participants.

**Participant**	**Subject ID**	**Age**	**Gender**	**BMI (kgm^-2^)**
1	1	30	M	30.00
2	5	30	M	24.75
3	8	27	M	19.32
4	9	25	M	21.70
5	12	32	F	20.20
6	16	24	M	16.66

### 1.2. Skin Conductance Deconvolution Using Compressed Sensing

#### 1.2.1. Skin Conductance Model Formulation

A skin conductance signal *y*_*SC*_(*t*) consists of two distinct parts. The comparatively slow varying part, also known as the tonic level, is primarily related to thermoregulation and is a function of ambient temperature and humidity. The other part, also known as the phasic component, fluctuates much faster and is generated by sympathetic nerve fibers stimulating the sweat glands. Therefore,

(1) $$\begin{array}{l} y_{SC}\left(t\right)=y\left(t\right)+y_{T}\left(t\right), \end{array}$$

where *y*(*t*) and *y*_*T*_(*t*) represent the constituent phasic and tonic components, respectively.

The phasic component *y*(*t*) can be extracted from *y*_*SC*_(*t*) using an algorithm such as cvxEDA (Greco et al., 2016). The physiology leading to the generation of the phasic component—namely the diffusion of sweat from the sweat duct to the stratum corneum, and its subsequent evaporation thereafter—can be modeled using first order dynamics (Alexander et al., 2005; Benedek and Kaernbach, 2010; Boucsein, 2012), and mathematically expressed via the following pair of differential equations:

(2) $$\begin{array}{l} {\dot{x}}_{1}\left(t\right)=-\frac{1}{\tau_{r}}x_{1}\left(t\right)+\frac{1}{\tau_{r}}u\left(t\right)\;\text{(diffusion)} \end{array}$$

(3) $$\begin{array}{l} {ẋ}_{2}\left(t\right)=\frac{1}{\tau_{d}}x_{1}\left(t\right)-\frac{1}{\tau_{d}}x_{2}\left(t\right)\;\text{(evaporation)} \end{array}$$

where *x*_1_(*t*) is an internal variable, *x*_2_(*t*) is the phasic component, and *u*(*t*) is the neural stimuli to the sweat glands. *x*_1_(*t*) is related to the amount of sweat and pressure within the sweat duct. The phasic component consists of a series of SCRs, each of which results from a single neural impulse burst. τ_*r*_ and τ_*d*_ are the rise and decay times of a single SCR.

The number of SCRs in a phasic skin conductance signal is typically much smaller than the total number of acquired data samples. Consequently, the number of underlying neural impulse bursts causing the SCRs is also small. This enables us to employ a sparsity constraint when solving for *u*(*t*). We model *u*(*t*) as a finite sum of weighted, shifted delta functions

(4) $$\begin{array}{l} u\left(t\right)=\overset{N}{\underset{i=1}{\sum }}u_{i}\delta \left(t-\Delta_{i}\right), \end{array}$$

where *u*_*i*_ represents the amplitude of an impulse occurring at Δ_*i*_, and *N* is the number of samples in the neural stimuli signal. *N* is proportional to the recording duration *T*_*d*_ and the input sampling frequency *f*_*u*_ (*N* = *T*_*d*_ · *f*_*u*_). Δ_*i*_ = *iT*_*u*_ where $T_{u}=f_{u}^{-1}$. *u*_*i*_ is positive if there is an impulse at time instance Δ_*i*_ and 0 otherwise. The continuous-time phasic skin conductance *y*(*t*) contaminated by measurement noise ν(*t*) is

(5) $$\begin{array}{l} y\left(t\right)=x_{2}\left(t\right)+\nu \left(t\right). \end{array}$$

If the signal is periodically sampled at *T*_*y*_ intervals to yield a total of *M* measurements, we can define the equivalent discrete-time observation *y*_*k*_ as

(6) $$\begin{array}{l} y_{k}=x_{2}\left(kT_{y}\right)+\nu_{k} \end{array}$$

where ν_*k*_ is Gaussian noise. Given all the discrete measurements *y*_*k*_ for *k* = 1, 2, …, *M*, we would like to recover *u*(*t*) and estimate τ_*r*_ and τ_*d*_. We take *x*_1_(0) = 0 as an initial condition assuming that the sweat duct is empty at the beginning. The state-space solution for *x*_2_(*kT*_*y*_) leads us to (Faghih et al., 2015b)

(7) $$\begin{array}{l} y_{k}=a_{k}y_{0}+{\text{b}}_{k}\text{u}+\nu_{k}, \end{array}$$

where $a_{k}=e^{-\frac{kT_{y}}{\tau_{d}}},{\text{b}}_{k}=[\frac{1}{\left(\tau_{r}-\tau_{d}\right)}\left(e^{-\frac{kT_{y}}{\tau_{r}}}-e^{-\frac{kT_{y}}{\tau_{d}}}\right)\;\frac{1}{\left(\tau_{r}-\tau_{d}\right)}\left(e^{-\frac{kT_{y}-T_{u}}{\tau_{r}}}-e^{-\frac{kT_{y}-T_{u}}{\tau_{d}}}\right)\;\frac{1}{\left(\tau_{r}-\tau_{d}\right)}\left(e^{-\frac{kT_{y}-2T_{u}}{\tau_{r}}}-e^{-\frac{kT_{y}-2T_{u}}{\tau_{d}}}\right)\;\cdot \cdot \cdot \;\frac{1}{\left(\tau_{r}-\tau_{d}\right)}\left(e^{-\frac{T_{u}}{\tau_{r}}}-e^{-\frac{T_{u}}{\tau_{d}}}\right)\;\underset{N-\frac{kT_{y}}{T_{u}}}{\underset{︸}{0\;\cdot \cdot \cdot \;0}}]$ and $\text{u}={\left[u_{1}\;u_{2}\;\cdot \cdot \cdot \;u_{N}\right]}^{⊤}$ represents a sparse vector containing all the neural stimuli over the entire signal duration (i.e., very few of the *u*_*i*_'s are non-zero). Concatenating all the measurements into a single vector $\text{y}={\left[y_{1}\;y_{2}\;\cdot \cdot \cdot \;y_{M}\right]}^{⊤}$ we have,

(8) $$\begin{array}{l} \text{y}={\text{A}}_{\tau }y_{0}+{\text{B}}_{\tau }\text{u}+\nu \end{array}$$

where ${\text{A}}_{\tau }={\left[a_{1}\;a_{2}\;\cdot \cdot \cdot \;a_{M}\right]}^{⊤}$, ${\text{B}}_{\tau }={\left[{\text{b}}_{1}^{⊤}\;{\text{b}}_{2}^{⊤}\;\cdot \cdot \cdot \;{\text{b}}_{M}^{⊤}\right]}^{⊤}$, $\nu ={\left[\nu_{1}\;\nu_{2}\;\cdot \cdot \cdot \;\nu_{M}\right]}^{⊤}$ and *y*_0_ is the initial condition of the phasic skin conductance signal. Here, *T*_*y*_ is an integer multiple of *T*_*u*_.

#### 1.2.2. Deconvolution

We set the sampling interval for the phasic skin conductance signal and neural stimuli to *T*_*y*_ = 0.5 s and *T*_*u*_ = 0.25 s, respectively. Equation (8) has *M* < *N* and represents an ill-posed problem with multiple solutions. The sparsity constraint on **u** however, makes it possible to solve the equation via compressed sensing. An *l*_1_-norm penalization term is typically added to the objective function to impose sparsity (Faghih, 2018). We consider *l*_*p*_-norm penalization in this particular formulation. We further constrain the rise and decay times to 0.1 ≤ τ_*r*_ ≤ 1.4 and 1.5 ≤ τ_*d*_ ≤ 6 similar to Amin and Faghih (2018, 2019). We impose these constraints based on prior work in the literature to ensure that the solution is identifiable and physiologically plausible (Alexander et al., 2005; Benedek and Kaernbach, 2010; Greco et al., 2016). Letting $\tau ={\left[\tau_{r}\;\tau_{d}\right]}^{⊤}$, we formulate the following constrained optimization problem based on Equation (8) to estimate **τ** and **u**

(9) $$\begin{array}{l} \underset{\begin{array}{c} \tau ,\text{u}\\ C\tau \leq b,\text{u}\geq 0 \end{array}}{\text{argmin}}J\left(\tau ,\text{u}\right)=\frac{1}{2}||\text{y}-{\text{A}}_{\tau }y_{0}-{\text{B}}_{\tau }\text{u}|{|}_{2}^{2}+\lambda ||\text{u}|{|}_{p}^{p}, \end{array}$$

where $C={\left[\begin{array}{cccc} -1 & 1 & 0 & 0\\ 0 & 0 & -1 & 1 \end{array}\right]}^{\text{T}}$, *b* = [−0.1 1.4 −1.5 6]^⊤^and λ is the *l*_*p*_-norm regularization parameter for imposing sparsity on **u**. λ is chosen to provide a balance between exploiting sparsity and accounting for signal fluctuations (Faghih, 2018). This optimization problem is challenging. We therefore decouple it into two sub-problems. A coordinate descent approach can be formulated similar to Faghih (2014, 2018), Faghih et al. (2014, 2015a,b) by solving the following sub-problems iteratively (for *l* = 0, 1, 2, ··· ) until convergence:

$${1.\text{u}}^{\left(l+1\right)}=\underset{\begin{array}{c} \text{u}\\ \text{s.t. }\text{u}\geq 0 \end{array}}{\text{argmin}}J_{\lambda }\left(\tau^{\left(l\right)},\text{u}\right)$$

$${2.\tau }^{\left(l+1\right)}=\underset{\begin{array}{c} \tau \\ \text{s.t. }C\tau \leq b \end{array}}{\text{argmin}}J\left(\tau ,{\text{u}}^{\left(l+1\right)}\right)$$

The first step represents a sparse recovery problem with a constrained convex optimization formulation. Many different approaches exist to solve this. One of the popular approaches is the iterative re-weighted least squares (IRLS) method. We solve this sparse recovery problem using two IRLS methods called FOCUSS+ (Murray, 2005) and GCV-FOCUSS+ (Zdunek and Cichocki, 2008). FOCUSS+ uses a heuristic approach for increasing λ at each IRLS step. We use FOCUSS+ for obtaining a suitable initialization of **u**. GCV-FOCUSS+ uses the Generalized Cross-Validation (GCV) technique to update λ at each step (Golub et al., 1979). We initialize GCV-FOCUSS+ with the result from FOCUSS+ and then run the IRLS until convergence. We finally constrain the minimum amplitude of any detected neural impulse to be 0.01 to reduce noisy detections. The second step in the coordinate descent approach represents a system identification problem with a constrained non-convex optimization formulation. We use the interior point method to solve this step.

The overall deconvolution algorithm begins by extracting the phasic skin conductance component using cvxEDA (Greco et al., 2016), then randomly initializing **τ** and performing the initialization step for **u** using FOCUSS+. Thereafter, we proceed with coordinate descent using GCV-FOCUSS+ and the interior point method. We perform the deconvolution on a small 3 min segment (taken from close to the opening portion of the experimental data we consider) of the signal to obtain the rise and decay times. Once we obtain these parameters, we use them to perform sparse recovery with GCV-FOCUSS+ on the entire skin conductance signal.

### 1.3. Sympathetic Arousal State Estimation

The autonomic nervous system contains both a sympathetic and a parasympathetic branch. The sympathetic branch mediates the body's “fight or flight" response and causes increases in blood pressure, perspiration and heart rate (Silverthorn, 2009). As pointed out earlier, sympathetic nerve fibers innervate the sweat glands (Low, 2012) and consequently skin conductance provides an index of sympathetic arousal (Critchley et al., 2002). Multiple skin conductance features such as tonic levels, rates of SCR appearance, SCR amplitudes and decay rates have been examined in the context of various behavioral interventions (Dawson et al., 2007; Boucsein, 2012). The rate at which SCRs occur has been shown to be related to cognitive task load (Jennings, 1986; Munro et al., 1987) and is thus a useful biomarker of autonomic arousal (Aikins et al., 2009). Here, we describe our approach of estimating an unobserved sympathetic arousal state based on the appearance of neural impulses underlying SCR generation.

We develop a state-space model relating arousal to the probability that the neural impulses occur (Wickramasuriya et al., 2018). The model is inspired by an earlier work relating a sequence of binary response variables to a latent cognitive learning state (Smith et al., 2004). We first divide the time-axis into bins of *T*_*u*_ duration indexed over *j* and assign *s*_*j*_ = 1 or *s*_*j*_ = 0 based on whether or not a neural impulse occurs at the *j*th time instance. Similar to Smith et al. (2004), we assume that sympathetic arousal *z*_*j*_ follows a random walk with time,

(10) $$\begin{array}{l} z_{j}=z_{j-1}+\epsilon_{j}\;;\;\epsilon_{j}\sim N\left(0,\sigma_{\epsilon }^{2}\right). \end{array}$$

The appearance of neural impulses *s*_*j*_ is a Bernoulli distributed random variable with probability *p*_*j*_ and is taken to be related to *z*_*j*_ via a sigmoid function (Smith et al., 2004),

(11) $$\begin{array}{l} \log\left(\frac{p_{j}}{1-p_{j}}\right)=\alpha +z_{j}\Rightarrow p_{j}=\frac{1}{1+e^{-(\alpha +z_{j}})}. \end{array}$$

The choice of the sigmoid function follows from the theory of generalized linear models (McCullagh and Nelder, 1989). Such logarithmic or exponential transformations are frequently encountered in count or frequency type data. Assuming that a subject's sympathetic arousal state *z*_0_ ≈ 0 at the outset of the experiment, $\alpha =\log\left[p_{0}{\left(1-p_{0}\right)}^{-1}\right]$ can be calculated by taking *p*_0_ as the probability that a neural impulse occurs randomly in a time bin for each individual (Smith et al., 2004; Wickramasuriya et al., 2018).

Given the observations *S*_1 : *J*_ = {*s*_1_, *s*_2_, …, *s*_*J*_} we wish to estimate *z*_*j*_ ∀ *j*. We use Bayesian filtering and Expectation-Maximization (EM) for estimating the arousal states *z*_*j*_ and recovering the unknown model parameters *z*_0_ and $\sigma_{\epsilon }^{2}$. The algorithm iterates between the E-step and M-step until convergence.

#### 1.3.1. Expectation Step

The E-step consists of two parts—a forward filter and a backward smoother. The filter first calculates a state estimate *z*_*j*|*j*_ using the observations *S*_1 : *j*_ available up to the *j*th time index. The backward smoother determines a second estimate *z*_*j*|*J*_ given all the available observations *S*_1 : *J*_. A Gaussian approximation is made at the filter formulation step and leads to the following equations at the *l*th EM iteration (Smith et al., 2004):

Predict:

(12) $$\begin{array}{l} z_{j|j-1}=z_{j-1|j-1} \end{array}$$

(13) $$\begin{array}{l} \sigma_{j|j-1}^{2}=\sigma_{j-1|j-1}^{2}+\sigma_{\epsilon }^{2\left(l\right)} \end{array}$$

Update:

(14) $$\begin{array}{l} z_{j|j}=z_{j|j-1}+\sigma_{j|j-1}^{2}\left[s_{j}-\frac{1}{1+e^{-(\alpha +z_{j|j}})}\right] \end{array}$$

(15) $$\begin{array}{l} \sigma_{j|j}^{2}={\left\{\frac{1}{\sigma_{j|j-1}^{2}}+\frac{e^{\alpha +z_{j|j}}}{1+e^{\alpha +z_{j|j}}{\;}^{2}}\right\}}^{-1}. \end{array}$$

One should note that *z*_*j*|*j*_ appears on both sides of Equation (14) and therefore is numerically solved using Newton's method. We next obtain the smoothed state and variance estimates *z*_*j*|*J*_ and $\sigma_{j|J}^{2}$ as follows (Mendel, 1995):

(16) $$\begin{array}{l} A_{j}=\frac{\sigma_{j|j}^{2}}{\sigma_{j+1|j}^{2}} \end{array}$$

(17) $$\begin{array}{l} z_{j|J}=z_{j|j}+A_{j}\left(z_{j+1|J}-z_{j+1|j}\right) \end{array}$$

(18) $$\begin{array}{l} \sigma_{j|J}^{2}=\sigma_{j|j}^{2}+A_{j}^{2}\left(\sigma_{j+1|J}^{2}-\sigma_{j+1|j}^{2}\right). \end{array}$$

#### 1.3.2. Maximization Step

We maximize the complete data likelihood at the M-step to estimate the two unknown model parameters $\sigma_{\epsilon }^{2}$ and *z*_0_. The parameter updates for the (*l* + 1)th iteration are as follows (Smith et al., 2004):

(19) $$\begin{array}{ll} \sigma_{\epsilon }^{2\left(l+1\right)}= & \frac{2}{J+1}[\overset{J}{\underset{j=2}{\sum }}\left(\sigma_{j|J}^{2}+z_{j|J}^{2}\right)-\overset{J}{\underset{j=2}{\sum }}\left(A_{j}\sigma_{j|J}+z_{j|J}z_{j-1|J}\right)] \end{array}$$

(20) $$\begin{array}{l} \;+\frac{1}{J+1}[\frac{3}{2}z_{1|J}^{2}+2\sigma_{1|J}^{2}-\left(\sigma_{J|J}^{2}+z_{J|J}^{2}\right)] \end{array}$$

(21) $$\begin{array}{ll} \;z_{0}^{\left(l+1\right)}= & \frac{1}{2}z_{1|J}. \end{array}$$

Following a criteria similar to Smith et al. (2004), we take the parameters to have converged once their absolute difference between consecutive iterations does not exceed 10^−8^.

#### 1.3.3. High Arousal Index

Similar to Smith et al. (2004), we calculate the probability that sympathetic arousal state *z*_*j*_ exceeds a specific threshold. We name this the High Arousal Index (HAI). HAI helps express how aroused a person is above a certain baseline. After the EM algorithm has converged, the state *z*_*j*_ at each time instance is taken to be Gaussian distributed $z_{j}\sim N\left(z_{j|J},\sigma_{j|J}^{2}\right)$ and we define HAI as follows:

(22) $$\begin{array}{l} \text{HAI}=\text{Pr}\left(z_{j}>z_{T}\right), \end{array}$$

where the threshold *z*_*T*_ is set to each subject's median state value across the whole experiment. Recall that the experiment acquired data from subjects during episodes of both stress and relaxation. The high stress induced during the experiment corresponds to a state of high arousal and the relaxation corresponds to low arousal. Therefore, we selected *z*_*T*_ as the median value as an approximation of normal arousal in between the two extremes.

## 2. Results

### 2.1. Skin Conductance Deconvolution

**Figure 2:**
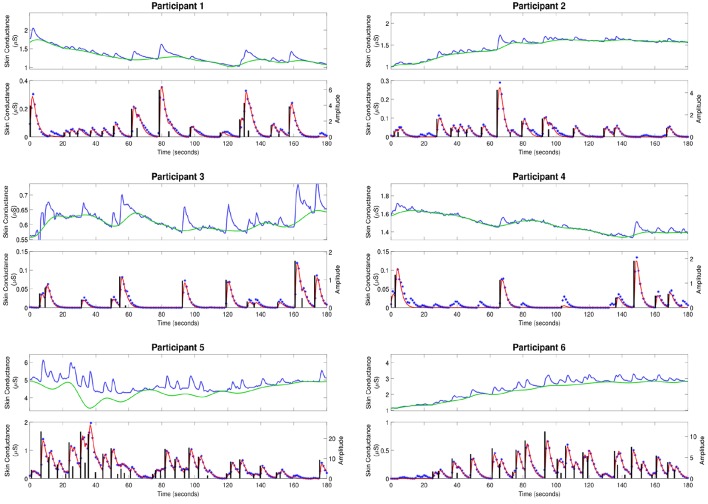
Estimated deconvolution of the experimental skin conductance data. For each of the participants, (i) the upper sub-panel depicts the raw skin conductance data (blue curve) and the separated tonic component of the skin conductance data using cvxEDA (green curve); (ii) the lower sub-panel depicts the separated phasic component (blue stars), the estimated reconstructed signal (red curve), the estimated neural stimuli timings and amplitudes (black vertical lines).

Figure 2 shows the skin conductance signals and deconvolution results for the selected participants during the backward counting task. In each sub-figure, the upper sub-panel shows the separation of the tonic and phasic components using cvxEDA (Greco et al., 2016). The lower sub-panel in each sub-figure shows the corresponding neural stimuli recovered using our deconvolution approach along with the reconstructed signal. It is the timings of these neural impulses that are used for estimating sympathetic arousal. Our method detects all significant impulses though it misses a few small ones that are comparable to noise. The number of detected impulses and the estimated SCR rise and decay times τ_*r*_ and τ_*d*_ are given in Table 2. Recall that these numbers are calculated based on data acquired during the backward counting task of the experiment. The τ_*r*_ and τ_*d*_ values estimated from this portion of the experiment are finally used to solve for the neural impulses over the entire signal. Also given in Table 2 are the squared multiple correlation coefficients *R*^2^ for the participants. *R*^2^ is an indication of goodness-of-fit and expresses how much of the variance of the data is captured by the model. *R*^2^ is above 0.93 for everyone indicating a good fit to the data. The number of impulses varies considerably from person to person. This is likely due to the fact that each participant responds to stress uniquely, despite being exposed to the same type of external stressor.

**Table 2:** Experimental results.

**Participant**	**τ_*r*_ (second)**	**τ_*d*_ (second)**	**∥u∥_0_**	**R^2^**
1	0.681	2.591	20	0.9629
2	1.398	1.568	17	0.9707
3	1.159	1.505	14	0.9868
4	0.965	1.880	8	0.9399
5	0.604	3.018	35	0.9788
6	0.663	2.617	28	0.9686

*The estimated model parameters and the squares of the multiple correlation coefficients (R^2^) for the fits of the experimental skin conductance time series*.

**Figure 3:**
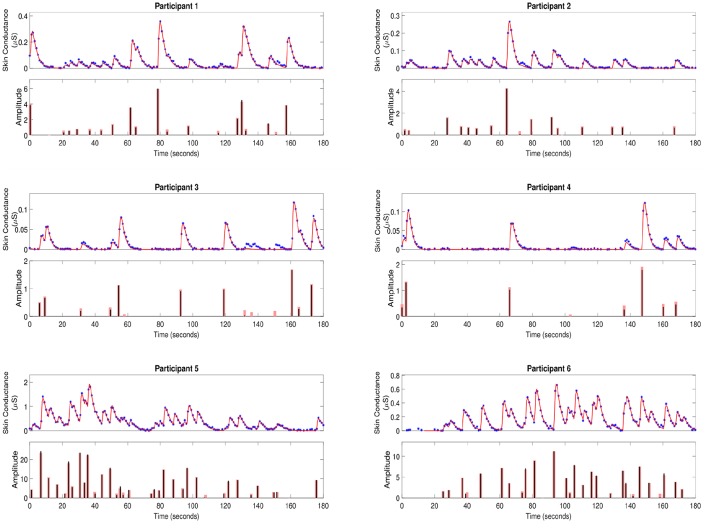
Estimated deconvolution of the simulated phasic skin conductance data in six participants. For each of the participants, (i) the upper sub-panel depicts the simulated phasic component of the skin conductance data samples with 25 dB SNR Gaussian noise (blue stars), the estimated reconstructed signal (red curve); (ii) the lower sub-panel depicts the estimated neural stimuli timings and amplitudes (black vertical lines) and the ground truth of the neural stimuli (red vertical lines) for each of the participants.

To further validate our deconvolution approach, we generated a second set of synthetic data using the **τ** and **u** already estimated for each participant. We added 25 dB SNR Gaussian noise to corrupt this new simulated phasic skin conductance data and performed deconvolution yet again. Figure 3 shows the results along with the ground truth. Table 3 shows the estimated parameters and their errors. Again, all *R*^2^ values are above 0.98 indicating a very good fit to the data.

**Table 3:** Results from simulated data.

**Participant**	**${\hat{\tau }}_{r}$ (second)**	**${\hat{\tau }}_{d}$ (second)**	**$||\hat{\text{u}}|{|}_{\text{0}}$**	**R^2^**	**$\frac{\left|{\hat{\tau }}_{r}-\tau_{r}\right|}{\tau_{r}}\times 100\%$**	**$\frac{\left|{\hat{\tau }}_{d}-\tau_{d}\right|}{\tau_{d}}\times 100\%$**	**$\left|||u|{|}_{0}-||\hat{u}|{|}_{0}\right|$**
1	0.664	2.627	19	0.9921	2.49	1.39	1
2	1.348	1.573	16	0.9919	3.58	0.32	1
3	1.128	1.503	11	0.9806	2.67	0.13	3
4	1.139	1.510	7	0.9891	18.03	19.68	1
5	0.5514	3.230	34	0.9936	8.71	7.02	1
6	0.650	2.672	27	0.9918	2.00	2.10	1

*The estimated model parameters and the squares of the multiple correlation coefficients (R^2^) for the fits of the experimental skin conductance time series*.

### 2.2. Sympathetic Arousal State Estimation

**Figure 4:**
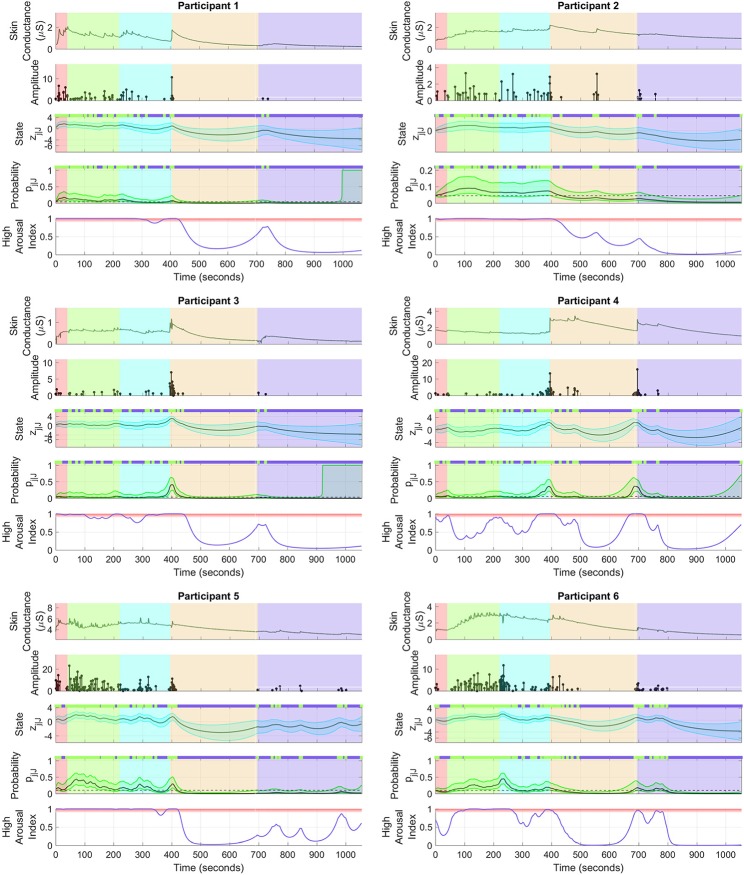
Sympathetic arousal state estimation. For each of the participants, (i) the top sub-panel depicts the skin conductance signal; (ii) the 2nd sub-panel depicts the recovered neural impulses; (iii) the 3rd sub-panel depicts the smoothed sympathetic arousal state *z*_*j*|*J*_ and its confidence intervals; (iv) the 4th sub-panel depicts the smoothed impulse occurrence probability *p*_*j*|*J*_ and its confidence intervals; (v) the lower sub-panel depicts the high arousal index (HAI) with the region above 90% probability highlighted in red. The color-coded backgrounds correspond to the instruction period for the cognitive tasks (red), the backward counting task (green), the Stroop test (cyan) (both the counting task and the Stroop test make up the cognitive stress portion), relaxation (light brown) and emotional stress (violet). Small green rectangles above 3rd and 4th sub-panels depict neural impulse location timings.

Figure 4 shows the sympathetic arousal state estimation results. For participant 1, arousal as measured by HAI remains above 90% during the cognitive tasks and reduces significantly during relaxation. HAI then increases around the start of emotional stress. Participant 2 has a similar response although the increase in arousal at the start of emotional stress is much less. There is also a notable, though not significantly high, increase right in the middle of the relaxation period. The arousal profile for participant 3 is almost identical to that of participant 1 with a high level at the start, a significant drop during relaxation and a moderate increase at the starting point of emotional stress.

The HAIs are somewhat different for the remaining participants. For participant 4, arousal increases up to or above the 90% threshold a few times during the cognitive tasks, but does not remain high continuously. There is a significant drop during relaxation and an increase above 90% when the horror movie begins. HAI for participant 5 remains high during the cognitive tasks and then drops during relaxation. There are several notable increases during emotional stress, though none of them increase above the 90% threshold. None of the increases however, exceed 90% in this period. Participant 6 is closest to participant 4 although HAI remains more consistently above 90% during cognitive stress with only a slight drop in the middle. Arousal thereafter drops during relaxation and increases above 90% for a brief period at the start of emotional stress. The estimated arousal states *z*_*j*|*J*_ also follow the general trend of the corresponding HAIs for all participants.

## 3. Discussion

### 3.1. Skin Conductance Deconvolution

Our method successfully recovers neural impulses associated with phasic SCRs. Between-subject variability in the estimated rise and decay times and the number of impulses is to be noted. The tonic levels also show considerable variations from person to person. These variations clearly highlight the need for determining the physiological parameters τ_*r*_ and τ_*d*_ on an individual basis rather than relying on fixed values for everyone.

Deconvolution using simulated data (Figure 3 and Table 3) also shows that most impulses are accurately recovered. Only impulses that are comparable to noise peaks are missed. The error percentages in estimating τ_*r*_ and τ_*d*_ are less than 10% for five of the participants. Participant 4 has a much higher percentage error. Recall that our problem formulation for estimating the rise and decay times is not convex. Consequently, there exists the possibility of stagnating at a local minimum. While we attempt to mitigate this problem through multiple initializations, and then taking the solution with the smallest squared error, there still exists a finite possibility of stagnating at a location other than the global minimum. The number of neural impulses for participant 4 is also much lower than for the others. Consequently, there are less SCRs to fit to and the result is more error-prone.

Each SCR, resulting from a single neural impulse, is mathematically modeled as a bi-exponential function. Estimating the rise and decay times of an SCR in the presence of noise is challenging due to this sensitive exponential nature. More than one pair of rise and decay times exist that can closely approximate an experimental SCR shape. Results are also heavily dependent on the removal of the tonic part. cvxEDA (Greco et al., 2016) models the tonic part with cubic B-spline basis functions with a 10 s knot size. Greco et al. (2016) used *l*_2_-norm penalization on the cubic spline basis function coefficients to avoid overfitting. They selected the regularization parameter to the penalization term based on prior data they had analyzed. This parameter depends on how the data is scaled in reality and how much of the tonic part it contains. Here, we set the regularization parameter related to the smoothness of the tonic component in cvxEDA at 0.001 instead of the 0.01 default to obtain a better separation of the tonic and phasic components.

Our current implementation of skin conductance deconvolution comprises of the two-step coordinate descent algorithm described earlier. Although it performs well in terms of accuracy, a faster implementation is necessary for a real-time CLDBS system. The physiological parameters τ_*r*_ and τ_*d*_ usually remain stable over a prolonged period of time. Therefore, we can perform only the sparse recovery procedure on windows of incoming data after an initial parameter estimation is complete, and thereafter estimate τ_*r*_ and τ_*d*_ in the background from time-to-time. A faster implementation of the sparse recovery step (e.g., using Greedy algorithms or Bayesian approaches) could further help improve time complexity.

### 3.2. Sympathetic Arousal State Estimation

A general trend is to be observed in the participant arousal levels in Figure 4. In the case of cognitive stress, the subjects' arousal states and HAI remain almost constantly high with the exception of participant 4. In contrast, there is only a moderate increase that dies down at the start of the emotional stress phase. The cognitive tasks required active engagement, i.e., mathematical calculations and active concentration, on the part of the participants. Meanwhile, the emotional stress period only involved passive engagement—the subjects just had to watch a horror movie clip without any significant cognitive effort. The varying level of cognitive activity is a likely reason for the difference in arousal between cognitive and emotional stress. Birjandtalab et al. (2016) used a clip from the horror movie entitled the “The Horde" to generate emotional stress. It is also possible that the movie was insufficient to generate significant emotional stress. The desired stress-generating effect may not have been realized if, for instance, a participant had already watched the movie. A visual inspection of the sub-panels depicting skin conductance in Figure 4 does not show a significant number of SCRs during emotional stress. It is likely PTSD patients may experience more emotional rather than cognitive stress. However, the emotions they experience may not necessarily be those evoked in healthy subjects for the very same stimuli. For instance, scenes of blood and dead bodies in a horror movie may evoke traumatic memories in PTSD patients leading to higher levels of stress. Further experimentation with a patient population would help validate our methods in detecting elevated levels of arousal in PTSD.

Sympathetic arousal information is not only encoded in how frequently neural impulses to the sweat glands occur, but also in the skin conductance signal amplitudes. Consequently, the amplitude of individual SCRs are taken as indicators of arousal (Bach et al., 2010). The tonic skin conductance level also contains emotion-related information (Braithwaite et al., 2013). Our current state-space formulation only considers the rate at which neural impulses (i.e., binary events) occur. Future work would incorporate the additional amplitude features for estimating sympathetic arousal using augmented state-space models that include both binary and continuous observations (Prerau et al., 2009; Coleman et al., 2011). The addition of heart rate could also help obtain an improved sympathetic arousal estimate (Wickramasuriya and Faghih, 2019).

The current EM approach is also offline and therefore requires modification if it is to be used in real-time in an experimental CLDBS prototype. We suggest running the forward filter in the E-step continuously and performing the full EM procedure in the background from time to time. This is very similar to the approach proposed for deconvolution when adapting to the needs of real-time computation. This may also permit the model parameters to change in adaption to disease progression and changing environmental conditions over time. The steps could also be run in parallel in a multicore processor. Several smartphones are now enabled with multicore processors and one option could be to perform the CLDBS computations on a wearer's phone. Another option would be to stream the data to the internet and perform computations in the cloud. Developing a custom hardware device to accompany the CLDBS implant is yet another option for performing skin conductance deconvolution and arousal state estimation in real-time.

### 3.3. Study Limitations

As noted earlier, many of the skin conductance recordings were contaminated with noise and had to be discarded. In-band motion artifacts such as those seen in the data-set usually contaminate a signal nonlinearly. Adaptive filtering (Mathews, 1991; Zaknich, 2005) and multi-level wavelet-based thresholding (Chen et al., 2015) are some of the options for suppressing motion artifacts. It is likely that skin conductance will indeed be contaminated with such artifacts in a real-world setting. We leave the development of an accelerometer-based adaptive filter for removing motion artifacts in skin conductance for future work. In another work, Amin and Faghih (2018) illustrated a way of performing concurrent deconvolution from multi-channel skin conductance data to obtain an estimate from noisy data. They included weights in different channels based on the standard deviation of noise in each channel while estimating the neural stimuli. They showed that the multi-channel approach could be more reliable for noisy skin conductance data. Multi-channel approach could potentially be used to achieve a more reliable CLDBS system. Moreover, factors other than sympathetic arousal can also influence skin conductance (e.g., body temperature, hydration, physical activity, and electrolytes). Variations induced by these factors may thus confound sympathetic arousal estimates; this is a limiting factor of using skin conductance alone. Such factors could be taken into account in a real-world setting and their effect canceled to obtain an improved arousal estimate. This would require an extended state-space model incorporating body temperature and hydration levels, for instance, as additional observations.

The present work is a proof-of-principle framework for using skin conductance in closing the DBS loop. As skin conductance relates to sympathetic arousal, and moreover as PTSD patients frequently show symptoms of hyperarousal, we note the suitability of using skin conductance as a CLDBS biomarker. The data-set used here however, does not include any PTSD patients and is a limitation of this study. Further investigation is therefore necessary to validate the use of skin conductance in an experimental CLDBS prototype for PTSD patients. Recall that differences in skin conductance have been reported in the literature between individuals with and without PTSD. The coefficients of the differential equations (**τ**) in Equations (2) and (3) governing sweat secretion dynamics are determined on a per subject basis. Thus, even if **τ** were significantly different between healthy and patient populations, our deconvolution methodology would still adapt to each individual. Furthermore, we only track a single skin conductance feature—the occurrence of neural impulses. The α coefficient in Equation (11) is calculated from the baseline probability of impulses on a per subject basis. The process noise variance $\sigma_{\epsilon }^{2}$ in Equation (10) is also estimated for each subject individually via the EM algorithm. Therefore, our Bayesian filter for sympathetic arousal estimation is also able to adapt to each individual. Although the present study did not include data from a patient population, we would however expect our methods to generalize to them nevertheless due to the ability of the framework to adapt to each individual.

### 3.4. Effect of DBS on Skin Conductance

The neural substrates underlying skin conductance have been examined in studies involving functional imaging, brain lesions and direct electrical stimulation (Critchley, 2002). Mangina and Beuzeron-Mangina (1996) applied electrical stimulation to the limbic structures of a group of subjects with intractable epilepsy and measured bilateral skin conductance. When the left sides of the amygdala, posterior hippocampus, anterior hippocampus and cingulate gyrus were stimulated, higher SCR amplitudes on the left hand were observed compared to the right. The reverse was also true when the right sides of the same interior structures were stimulated. They also reported that stimulation intensity increased SCR amplitudes. Lanteaume et al. (2006) examined the effect of electrical stimulation of the amygdala on self-reported emotions and SCRs in a group of patients with drug-resistant partial epilepsy. SCR amplitudes were larger when the stimulation caused a positive emotional change as opposed to a negative change or no change. Our methodology does not make use of the SCR amplitudes but rather the rates at which they occur. Further research would be necessary to quantify the effect on SCR rates when applying direct stimulation via a DBS implant to the limbic structures. A correction for the effect due to the stimulation could be incorporated into the state-space model in this case.

### 3.5. Closing the DBS Loop

Developing a complete CLDBS system is a challenge. Our approach demonstrates the ability to recover sympathetic arousal from skin conductance measurements using state-space methods. Neural stimuli to the sweat glands originating from the sympathetic nerve fibers encode emotion-related information in how frequently they fire. By relating the probability of neural impulse occurrence to sympathetic arousal through a state-space model, we are able to estimate a continuous state trajectory across different episodes of relaxation and stress.

Different biomarkers have been suggested for closing the loop in DBS therapy. Ideally, the biomarker should enable the real-time tracking of an unobserved brain state. Here, we investigate skin conductance as a viable alternative for a CLDBS as a means of treating neuropsychiatric disorders such as PTSD. While we have sought to address how to estimate an emotion-related state trajectory from peripheral skin conductance measurements, there remains the problem of determining the mapping from the state variable back into the CLDBS stimulation parameter space (i.e., the amplitude, frequency, and width of the electrical stimuli). Determining this mapping will enable sympathetic arousal to be controlled in realtime. Grahn et al. (2014) proposed a novel means of addressing this mapping problem in a CLDBS system they developed for maintaining stable dopamine levels in rodents. They first varied the frequency, amplitude, and pulse width of the electrical stimulation and measured the corresponding dopamine level responses for different parameter combinations. We too could similarly vary the electrical stimulation parameters of a DBS system while measuring skin conductance changes and estimate the corresponding arousal levels. Grahn et al. (2014) next characterized the dopamine responses using a combination of 7th order polynomials and 2nd order exponentials. This required a total of 12 model coefficients. They next trained a neural network having the frequency, amplitude and pulse width as the inputs and the 12 model coefficients as the outputs. Likewise, we could train a neural network mapping stimulation parameters to arousal responses. Thereafter, Grahn et al. (2014) trained a *second* neural network having the model coefficients as inputs and the frequency, amplitude and pulse width as the outputs. They used this inverse model for predicting the stimulation parameters necessary for maintaining specific extra-cellular dopamine levels. Therefore, a CLDBS system utilizing our method for arousal estimation could utilize a similar neural network characterizing the inverse relationship back into the stimulation parameter space in its feedback path.

A simpler option would be to use on/off control instead of adjusting the stimulation parameters in a continuous manner. Herron et al. (2017) developed an on/off CLDBS controller for a patient suffering from tremor. The controller applied electrical stimulation when β-band power recorded from invasively acquired electroencephalography (EEG) dropped below a certain (manually-tuned) threshold, and switched it off when the power exceeded yet another threshold. Preliminary on/off control could be applied in the case of neuropsychiatric disorders too, for instance when sudden angry outbursts or bouts of depression as detected by abnormally elevated or diminished arousal levels occur.

We wish to point out that not all elevated arousal levels need to be controlled (e.g. high arousal levels due to positive excitement). When proposing a CLDBS framework for the treatment of chronic pain, Shirvalkar et al. (2018) too noted that they did not wish to avoid all feelings of pain *per se*, as pain itself provides a warning of potential tissue damage. Therefore, a fully closed-loop system for the treatment of neuropsychiatric disorders should be able to recognize the positive–negative aspect of emotion as well. This positive–negative or pleasure–displeasure dimension is known by the term emotional valence (Russell, 1980). The CLDBS would ideally need to estimate a vector **z**_*j*_ containing both arousal and valence. Emotional valence information can be decoded using physiological signals such as scalp EEG, heart rate, electromyography and skin conductance (Koelstra et al., 2012; Soleymani et al., 2012). Stimulation can then be turned on, for instance, only if high arousal is detected and valence is negative.

Finally a control algorithm is necessary in the CLDBS feedback loop when progressing beyond preliminary on/off stimulation. Our state estimation approach relies on a rate of neural firing. Azgomi et al. (2019) developed a fuzzy feedback controller based on the rate of SCR appearances for increasing arousal during relaxation and decreasing it during periods of cognitive stress. The controller was based on the model proposed in Wickramasuriya et al. (2018) and can directly be used with the method presented here. In a review of CLDBS therapy, Carron et al. (2013) mention two different other works, namely those by Grant and Lowery (2013) and Pasillas-Lépine et al. (2013), that propose a control mechanism based on the rate of neural firing for estimating a state variable. Pasillas-Lépine et al. (2013) proposed a proportional control based on the firing rates of the subthalamic nucleus and global pallidus. Grant and Lowery (2013) developed a similar CLDBS controller based on β-band oscillations in LFPs. An adaptation of one of these methods could also be used for developing a control law for regulating emotion based on peripheral skin conductance measurements.

### 3.6. Conclusion

DBS has met with success in treating a host of disease conditions where other therapeutic measures have been exhausted. CLDBS systems have been proposed as the future of DBS due to their inherent advantages over the previous generation of open-loop systems. Closing the DBS loop is a challenge. In this work, we present a method for estimating sympathetic arousal from skin conductance measurements as a potential mechanism that could be deployed within a CLDBS system for treating neuropsychiatric disorders. The methodology consists of two parts: (i) the deconvolution of phasic skin conductance to obtain the neural impulses that generate SCRs; (ii) a state-space model for tracking sympathetic arousal based on the frequency at which the SCRs appear. Results are demonstrated on a publicly available data-set. We finally discuss possibilities for developing a controller that could map the state estimates back into the stimulation parameter space for automated closed-loop control. While we mention PTSD as an example scenario here, our approach could be generalized to other disease conditions where any type of impulse-like or pulsatile signal is a biomarker. For instance, if neural spiking or pulsatile cortisol secretions are clinically-relevant features for a particular disease condition, then the Bayesian filter described here could also be used as part of closing the loop. Additionally, the methods presented here are personalized, i.e., the model parameters are estimated for each individual. Differences in skin conductance have been reported in the literature between healthy subjects and patients with anorexia nervosa (Tchanturia et al., 2007) and depression (Ward and Doerr, 1986). If skin conductance biomarkers can be determined for each of these conditions (based on SCR amplitudes, rates of SCR occurrence, skin conductance levels etc.), then an extended state-space model could be developed to track a symptom-related neural state that incorporates both binary and continuous-valued observations (Prerau et al., 2009; Coleman et al., 2011).

## Data Availability

Publicly available datasets were analyzed in this study. This data can be found here: https://physionet.org/physiobank/database/noneeg/.

## Author Contributions

RF conceived and designed the study. DW and MA analyzed data and wrote the manuscript. RF, DW, and MA developed the algorithms and analysis tools.

### Conflict of Interest Statement

The authors declare that the research was conducted in the absence of any commercial or financial relationships that could be construed as a potential conflict of interest.
